# Ultralow-dose Dexamethasone to Preserve Endogenous Cortisol Stress Response in Nonclassical Congenital Adrenal Hyperplasia: A New Promising Treatment

**DOI:** 10.5812/ijem.14657

**Published:** 2014-07-01

**Authors:** Danielle van der Kaay, Erica van den Akker

**Affiliations:** 1 Department of Pediatric Endocrinology, Erasmus Medical Centre, Rotterdam, The Netherlands

**Keywords:** Adrenal Hyperplasia, Congenital, Dexamethasone, Therapeutics, Hyperandrogenism

## Abstract

**Introduction:**

Nonclassical congenital adrenal hyperplasia (CAH) is characterized by sufficient cortisol and aldosterone production at the cost of androgen overproduction. Hydrocortisone or dexamethasone in supraphysiological doses are current treatment; however, their downside is suppression of endogenous cortisol production resulting in corticosteroid dependency. We aimed to treat children with nonclassical CAH with a ultralow-dose dexamethasone to normalize androgen levels, without a detrimental effect on endogenous cortisol production.

**Case Presentation:**

We recruited five patients diagnosed with nonclassical CAH on the basis of clinical presentation, biochemical analyses, and genetic testing. Anthropometric as well as biochemical parameters and bone age were measured on a regular basis. During treatment, an adrenocorticotropin (ACTH) stimulation test was performed. Outcome measures were normalization of androgens and deceleration of the bone age advancement with sufficient endogenous cortisol response. Androgen levels were normalized in all patients resulting in a deceleration of the bone age advancement. Cortisol stress response remained normal in four out of five patients. Only one patient needed hydrocortisone stress dosing.

**Conclusions:**

According to this case series, it seems that ultralow-dose dexamethasone in treatment of nonclassical CAH would be a promising novel treatment strategy. The advantage of this treatment strategy is that adverse effects of hyperandrogenism can be reversed while preserving the endogenous cortisol stress response.

## 1. Introduction

Nonclassical CAH is characterized by sufficient cortisol and aldosterone production at the cost of androgen overproduction. In 90% to 95% of cases, CAH is caused by mutations in the CYP21A2 gene, resulting in 21-hydroxylase enzyme deficiency (1). Children with nonclassical CAH present with precocious pubarche, hirsutism, and advanced bone age without a clinically significant accelerated growth, which results in short adult height (2). In children with nonclassical CAH and premature pubarche without advanced bone age, treatment can be withheld under careful observation. In case of advanced bone age or severe symptoms of hyperandrogenism, children are usually treated with hydrocortisone replacement therapy (10-15 mg/m^2^/day) (3). Treatment goal is to replace cortisol with a synthetic glucocorticoid that will suppress ACTH overproduction and normalize hyperandrogenism; however, the downside of this treatment is suppression of endogenous cortisol production that makes the child dependent on synthetic glucocorticoids.

Observations made more than 50 years ago as well as more recent clinical studies demonstrated that dexamethasone is probably 70 to 100 times more potent than hydrocortisone in suppressing androgen production (4, 5). Based on these observations, we hypothesized that treatment with the ultralow-dose dexamethasone of 0.025 mg at night would result in normalization of androgen levels without any detrimental effect on endogenous cortisol production.

## 2. Case Presentation

We recruited five patients diagnosed with nonclassical CAH based on clinical presentation, biochemical analyses, and genetic testing. An ACTH stimulation test using 0.25 mg ACTH was performed in all patients. A 60-minute stimulated 17-hydroxyprogesterone (17-OHP) level higher than 30 nmol/L (1000 ng/mL) was considered diagnostic for nonclassical CAH. Diagnosis was confirmed by CYP21A2 gene mutation analysis in patients with the typical clinical phenotype, elevated androgen levels, advanced bone age (> 2 years), and a peak stimulated 17-OHP level of higher than 10 nmol/L. Analysis using Sanger sequencing of all coding exons and flanking intron-exon boundaries as well as deletion and duplication analysis of the CYP21A2 gene by multiplex ligation-dependent probe amplification (MLPA) were performed at the Radboud University Medical Center in Nijmegen, the Netherlands. 

Patients visited the outpatient clinic every three months. Height was measured using a Harpenden stadiometer and expressed as the standard deviation score (SDS) for the respective calendar age (6). Saliva was collected every six weeks until 17-OHP and androstenedione levels stabilized. After stabilization of 17-OHP, saliva samples were collected every three months. Venapuncture for determining the serum 17-OHP and androstenedione levels was performed on the annual basis. When salivary 17-OHP and androstenedione levels were above the cut-off value of 0.5 and 0.25 nmol/L, respectively, dexamethasone dose would be increased. Similarly, when serum 17-OHP and androstenedione levels were above the age-appropriate range, dexamethasone dose would be increased. Within six months of treatment, an ACTH test was performed to investigate the adrenal stress response during dexamethasone treatment. Dexamethasone treatment was continued the night before testing. Cortisol levels above 500 nmol/L were considered as sufficient response. In that case, no hydrocortisone stress dosing was initiated in case of severe illness, major trauma, or surgery. Bone age was determined on the annual basis using the Greulich-Pyle method.

All patients presented with precocious pubarche and an advanced bone age of +1.5 to +3.8 years at the start of treatment (Table 1). Patients had signs of precocious pubarche such as pubic hair and/or axillary hair, acne and/or body odor without signs of increased growth velocity up to three years before presentation. ACTH stimulated 17-OHP levels were above 30 nmol/L in four out of five patients. In patient 2, genetic testing demonstrated a heterozygous V281L mutation. Treatment was started after two years of follow-up because of increasing clinical signs of androgen overproduction including an advanced bone age of almost four years. 

All patients were naïve to glucocorticoid treatment before start of treatment with 0.025 mg dexamethasone at night (start dose, 0.019-0.026/m^2^/day). In patients 1, 2, 4 and 5, dexamethasone dose was increased to 0.022-0.032 mg/m^2^/day after 18 to 36 months due to salivary and/or serum 17-OHP and androstenedione levels being above the reference range. All patients, except patient 1, had sufficient cortisol response (> 500 nmol/L) during the 1 μg ACTH test. For patient 1, cortisol response was borderline and insufficient; hence, hydrocortisone stress dosing was advised in case of severe illness, major trauma, or surgery. 

During treatment, the difference between bone age and chronological age decreased substantially in all patients. Moreover, bone age was similar to chronological age (≤ 1 year difference) in three patients (Table 1).

**Table 1.** Anthropometric and Laboratory Characteristics of Patients ^a^

Variables	Patients ID 1	Patients ID 2	Patients ID 3	Patients ID 4	Patients ID 5
	Male	Male	Female	Female	Male
**Age at Presentation, y**	9.2	6.1	7.7	6.1	8.8
**Bone Age at Presentation, y**	10.8	9	9.8	6.8	12.3
**Stimulated 17-OHP, nmol/L**	127	13	96	127	30
**Stimulated Cortisol, nmol/L**	524	840	830	601	1138
**DNA Analysis**	V281L/V281L	V281L/WT1	V281L/V281L	V281L/V281L	V281L/WT
**Age at Start Treatment, y**	9.9	8.1	8.3	7.9	10.8
**Target Height, SDS**	+0.1	-0.5	-0.3	-1.1	0
**Dexamethasone Dose, mg/m** ^**2**^ **/day**	0.021	0.019	0.026	0.026	0.019
**Height, SDS**	0.0	+1.0	-0.2	-0.6	-0.4
**BA-CA, y**	+1.7	+3.8	+2.1	+2	+3.5
**BMI, SDS**	+0.4	+2.8	-1.3	+0.4	+1.7
					
**Duration of Follow-up, y**	4.8	2.8	1.7	1.7	4.8
**Dexamethasone Dose, mg/m** ^**2**^ **/day**	0.027	0.032	0.023	0.032	0.022
**Height, SDS**	-0.5	+0.6	-0.7	-0.4	-0.5
**BA-CA, y**	-1	+1	+1	+1.4	+1.3
**BMI, SDS**	-0.8	+2.6	-0.5	+0.9	+2.5
**Stimulated Cortisol, nmol/L**	356	720	555	524	579

^a^ Abbreviations: 17-OHP, 17-hydroxy progesterone; SDS, standard deviation score; BA, bone age; CA, chronological age; BA-CA, the difference between bone age and chronological age; BMI, body mass index; WT, wild type.

## 3. Discussion

Ultralow-dose dexamethasone at night resulted in normalization of androgen levels in our patients including three patients with nonclassical CAH and two symptomatic carriers with the clinical phenotype of nonclassical CAH. The process of the bone age advancement showed a substantial deceleration, resulting in a comparable bone age with calendar age in three patients. The endogenous cortisol stress response remained normal in four out of the five patients. 

To the best of our knowledge, this is the first case series describing children with nonclassical CAH who were treated with ultralow-dose dexamethasone. Treatment with dexamethasone in children with simple virilizing CAH was reported by Rivkees et al. with promising results (7-8). The average daily morning dose in the first study was 0.27 mg/m^2^/day. These children started treatment at an average age of 2.8 years. Adult height or projected adult height SDS of most patients was between +0.5 and -1.0 (7). In a second study, children with classical simple virilizing CAH were treated from birth onwards with a mean daily dose of 0.18 ± 0.5 mg/m^2^/day. After 6.5 years follow-up, these children had normal growth velocities (mean z score, 0.5 ± 0.2) and appropriate bone age (bone age to chronological age ratio, 0.9 ± 0.6 years) (8). Growth of our patients was normal and within the target height range. Furthermore, the difference between bone age and chronological age decreased substantially in all patients, and bone age was comparable to chronological age after several years of treatment in three patients. 

Recently, Matthews and Cheetham have addressed the question whether teenagers presenting with nonclassical CAH should always be treated with glucocorticoids (9). Generally, in children with nonclassical CAH and precocious pubarche without advanced bone age, treatment can be withheld under careful observation. The authors stated that the adult height in patients with nonclassical CAH was not compromised and referred to a study performed in 12 patients between three and 33 years of age. Eight patients aged between two and nine years of age. However, nine out of 12 patients were treated with glucocorticoids, which would most likely have resulted in a substantial decrease of bone age advancement (10). Progressive advancement of bone age will result in premature closure of epiphyseal growth plates that will compromise adult height. Studies investigating adult height in untreated patients with nonclassical CAH are very scarce. Adult height of untreated patients with nonclassical CAH seems to be around 8.5 cm lower in comparison to treated patients (162.9 ± 12.9 cm vs. 171.4 ± 14.9 cm) (11). We demonstrated that treating children with nonclassical CAH with ultralow-dose dexamethasone diminishes bone age advancement. This is expected to influence adult height towards taller stature. 

This clinical case series has several limitations. In this pilot study, only a small number of patients were treated. Two of our patients were heterozygous for the V281L mutation; a previous study showed that symptomatic carriers for the V281L mutation have significantly higher stimulated 17-OHP levels in comparison to asymptomatic carriers and carriers for other mutations (12). One hypothesis is that impairment of enzymatic activity in these symptomatic carriers is caused by a dominant-negative effect of this mutant allele. Another explanation is that these children carry another unidentified mutation on the alternate allele. It is unclear whether these two overweight male patients had premature adrenarche rather than nonclassical CAH. Heterozygous V281L mutations have been described in patients with premature adrenarche (13). Since bone age was advanced almost four years in both patients, we decided to treat these patients with ultralow-dose dexamethasone. The follow-up period varied from 18 month to almost five years. Although the final adult height result could not be exactly determined, two of our study children reached a near adult height within the target height range. Our results are preliminary and long-term data and larger randomized studies have to be performed before drawing any definite conclusions.

In conclusion, this case series suggests that ultralow-dose dexamethasone might be a promising new treatment strategy in children with nonclassical CAH through normalization of hyperandrogenism and a less advanced bone age while preserving endogenous cortisol response. In line with the article of Matthews and Cheetham, the question of “what is the best glucocorticoid regimen in patients with CAH” still has to be answered. We speculate that patients with nonclassical CAH, especially those with a relatively mild enzyme defect, can benefit from treatment with ultralow-dose dexamethasone.
